# Post-Operative Superior Mesenteric Artery Syndrome Following Retroperitoneal Sarcoma Resection

**DOI:** 10.3390/clinpract11010002

**Published:** 2020-12-24

**Authors:** Liam H. Wong, Thomas L. Sutton, Ryan G. Spurrier, Andrew F. Zigman, Skye C. Mayo

**Affiliations:** 1 School of Medicine, Oregon Health & Science University (OHSU), 3181 SW Sam Jackson Park Rd, Portland, OR 97239, USA; wonli@ohsu.edu; 2 Division of Surgical Oncology, Oregon Health & Science University (OHSU), 3181, SW Sam Jackson Park Rd, Portland, OR 97239, USA; suttoth@ohsu.edu; 3 Division of Pediatric Surgery, Oregon Health & Science University (OHSU), 3181 SW Sam Jackson Park Rd, Portland, OR 97239, USA; rspurrier@chla.usc.edu (R.G.S.); andrew.f.zigman@kp.org (A.F.Z.); 4 Division of Pediatric Surgery, Children’s Hospital of Los Angeles, 4650 Sunset Blvd, Los Angeles, CA 90027, USA; 5 OHSU Knight Cancer Institute, 3181 SW Sam Jackson Park Rd, Portland, OR 97239, USA

**Keywords:** superior mesenteric artery syndrome, SMA syndrome, retroperitoneal sarcoma, duodenal obstruction, gastrointestinal symptom

## Abstract

Superior mesenteric artery (SMA) syndrome is an uncommon phenomenon caused by the compression of the third portion of the duodenum between the aorta and the SMA. Here, we present a previously healthy 15-year-old male who presented with early satiety and 20 kg weight loss. Computed tomography (CT) demonstrated a massive retroperitoneal liposarcoma displacing the entire small intestine into the right upper quadrant. Following resection of the large mass, the patient was intolerant of oral intake despite evidence of bowel function. Abdominal CT revealed a narrowing of the duodenum at the location of the SMA. A nasojejunal feeding tube was placed past this area, and enteral nutrition was initiated before slowly resuming oral intake. Post-operative SMA syndrome is an uncommon complication but should be considered in patients intolerant of oral intake following resection of large abdominal tumors associated with extensive retroperitoneal fat loss, even in the absence of concomitant major visceral resection.

## 1. Case Report

**Figure 1:**
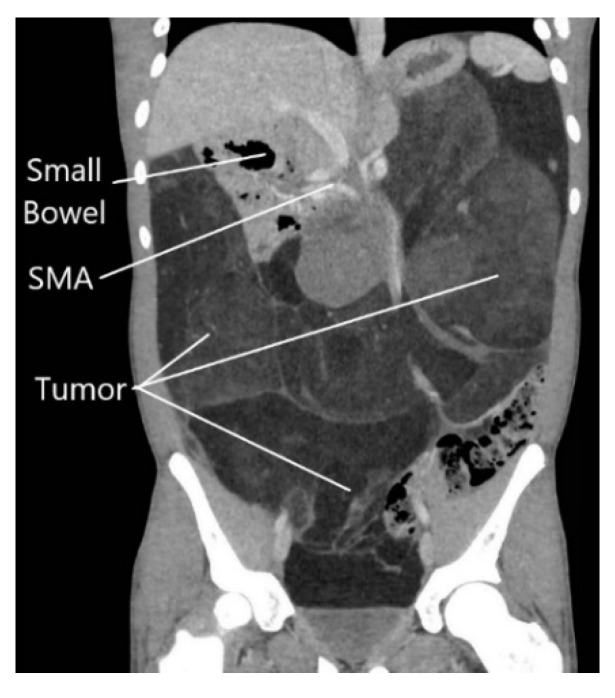
Abdominal computed tomography scan showing massive retroperitoneal liposarcoma, with displacement of the small intestine to the right upper quadrant.

A previously healthy 15-year-old male was referred to our hospital presenting with early satiety, 20 kg weight loss (body mass index = 18.1 kg/m^2^), and abdominal fullness on exam. Abdominal computed tomography (CT) revealed a massive retroperitoneal tumor shifting the entire small bowel into the right upper quadrant, with significant lateral displacement of the superior mesenteric artery (SMA) from its normal anatomic course (Figure 1). The tumor was compressing all intra-abdominal organs, including the stomach, likely causing his symptoms of early satiety. Percutaneous biopsy of the mass demonstrated a well-differentiated liposarcoma. Chest CT did not show any evidence of lung metastases.

**Figure 2:**
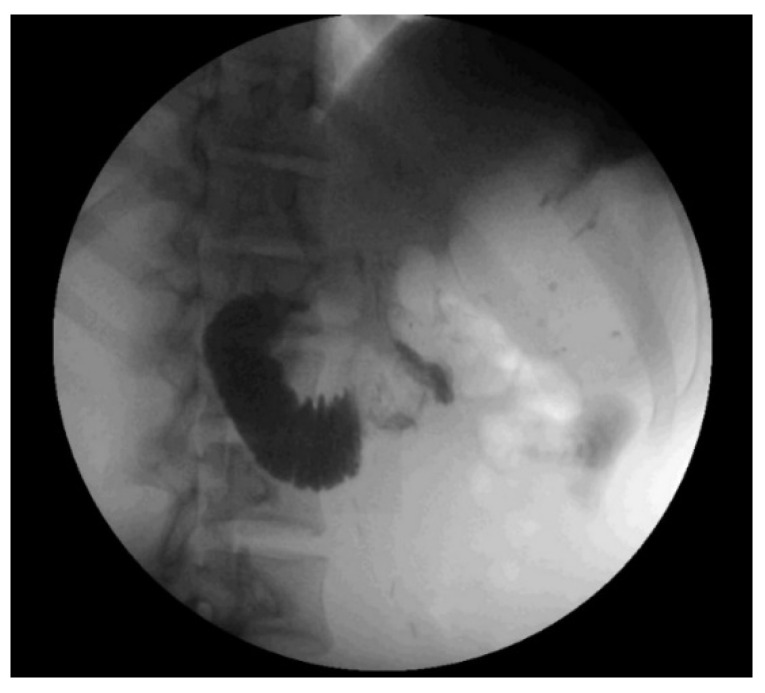
Representative image from upper gastrointestinal series showing abrupt cutoff of oral contrast in the mid-transverse duodenum, suggestive of superior mesenteric artery syndrome.

**Figure 3:**
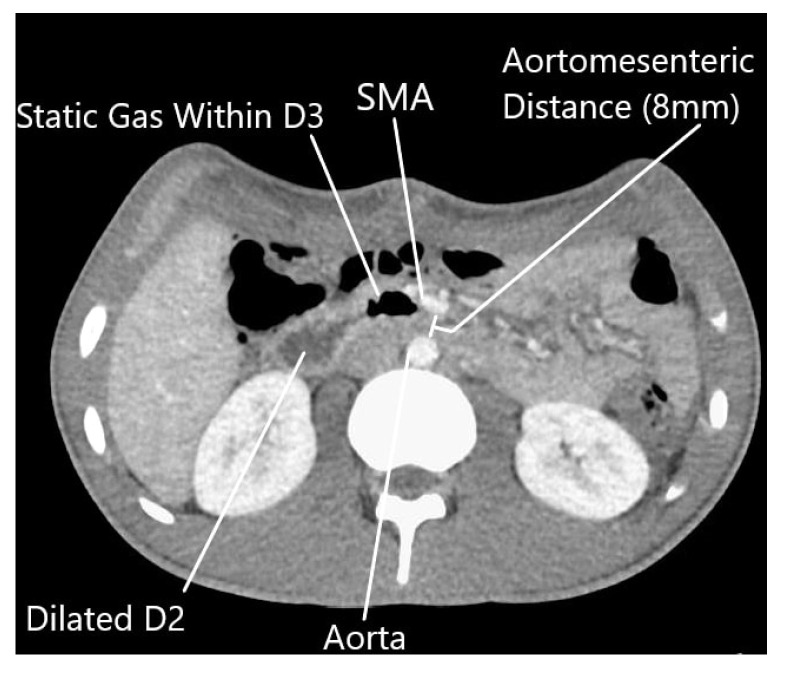
Abdominal computed tomography scan showing post-operative return of superior mesenteric artery to normal anatomic position, with reduced aortomesenteric distance (8 mm). (SMA—superior mesenteric artery, D2—second portion of the duodenum, D3—third portion of the duodenum).

The patient underwent resection of the 11.1 kg, 70 cm mass without *en bloc* visceral resection; surgical margins were negative with no capsular disruption or intra-operative tumor spillage. It is worth noting that the duodenum was visualized crossing the midline, and the ascending, transverse, and descending colons were in their normal anatomic positions, excluding pre-existing malrotation. At the conclusion of the resection, he was noted to have near-complete absence of lipomatous tissue in his retroperitoneum. Post-operatively, he was persistently intolerant of diet advancement; despite apparently successful attempts at nasogastric decompression with evidence of bowel function, he demonstrated persistent bilious emesis with a benign abdominal exam. An upper gastrointestinal series revealed abrupt cutoff of contrast in the mid-duodenum, with significantly delayed transit of contrast distally (Figure 2). Abdominal CT demonstrated duodenal compression by the SMA with a reduced aortomesenteric distance of 8 mm, diagnostic of SMA syndrome (Figure 3). A nasojejunal feeding tube was placed fluoroscopically; the patient quickly tolerated goal tube feeds and began gaining weight. He remained *nil per os* for 4 weeks after discharge with continued weight gain, before slowly resuming oral intake. The patient’s intolerance to oral intake has resolved; he is now independent of tube feedings and has returned to near the 50th weight percentile for his age and height.

## 2. Discussion

Superior mesenteric artery syndrome is an uncommon phenomenon with an estimated incidence in the general population between 0.013% and 0.3% [1,2,3]. SMA syndrome is defined as the compression of the third portion of the duodenum between the aorta and the SMA, leading to a functional point of obstruction. The SMA arises from the abdominal aorta at the first lumbar vertebral body level, and typically maintains an angle of 38 to 65 degrees relative to the aorta, with an aortomesenteric distance of 10 to 28 mm, between which the duodenum traverses [4]. The root cause of SMA syndrome is narrowing of this distance, causing compression of the duodenum [2,4].

Patients with SMA syndrome predominantly present with a history of chronic abdominal complaints characterized by signs and symptoms of duodenal obstruction but varying due to the degree of duodenum compression [3]. Symptoms can range from postprandial pain and nausea to complete bowel obstruction symptoms [1]. Diagnosis is based on clinical evidence of intolerance of oral intake with diagnostic imaging to show obstruction at the third portion of the duodenum [1]. Upper gastrointestinal series classically show gastric and proximal duodenal dilation, with an abrupt termination of the contrast material column in the third portion of the duodenum [3]. CT and magnetic resonance scans allow visualization of the site of duodenal obstruction relative to the SMA and precise measurement of the aortomesenteric angle and distance [1,3]. CT criteria for the diagnosis of SMA syndrome include an aortomesenteric angle of less than 22 degrees or an aortomesenteric distance of less than 8 to 10 mm [5].

Epidemiologically, females are more commonly affected, with two-thirds of patients 10 to 40 years of age [1]. Risk factors for developing SMA syndrome fall into three categories: severe rapid weight loss, external and intra-abdominal compression, and mesenteric tension [3]. SMA syndrome is most commonly found in rapid weight loss with wasting of retroperitoneal adipose tissue that bolsters the aortomesenteric distance as seen in our patient. Rapid weight loss and wasting conditions can lead to the loss of this retroperitoneal fat tissue, resulting in the narrowing of the aortomesenteric angle [2,6]. This phenomenon has been observed in the setting of AIDS [7], cancer [8,9], eating disorders [4], cerebral palsy [4], substance abuse [10], and other catabolic and malabsorptive states [11].

Post-operative SMA syndrome following intra-abdominal procedures is extremely rare, but has previously been reported following proctocolectomy and ileoanal pouch anastomosis [12], Nissen fundoplication [13], and aortic aneurysm repair [14]. Corrective spinal surgery for scoliosis, which requires relative lengthening of the spine and results in the narrowing of the aortomesenteric angle, is the most frequently cited cause of post-operative SMA syndrome with an estimated incidence of 2.5% [7,15]. Previously, only one instance of post-operative SMA syndrome following an intra-abdominal mass resection has been reported, occurring following enucleation of a 7 cm teratoma at the uncinate process of the pancreas [16]. This may have been due to altered local anatomy given the proximity of the uncinate process to the SMA. To our knowledge, the case we have presented is the first reported incidence of SMA syndrome occurring following resection of a large retroperitoneal tumor without visceral resection.

In the present case, the patient’s large intra-abdominal tumor was displacing the entire small bowel to the right upper quadrant, effectively “propping open” the aortomesenteric angle as seen by the perpendicular course of the SMA compared with normal anatomy (Figure 1). The patient’s pre-operative weight loss, coupled with a rapid return of the small bowel mesentery to its normal anatomic position, likely resulted in the observed SMA syndrome. Another unique feature distinguishing this case from other reported post-operative SMA syndrome cases is that apart from an appendectomy, no native anatomic structures were resected or artificially rearranged in an effort to obtain negative surgical margins.

Lam et al. assessed the clinical symptoms for SMA syndrome following surgical treatment of scoliosis and presented an algorithm on how to treat SMA syndrome [17]. Initial treatment consists of nasogastric decompression and correction of electrolytes and intravenous hydration, followed by enteral nutrition through nasojejunal tube or parenteral nutrition if necessary [3,10,17]. These measures aim to increase body weight to promote the restoration of the retroperitoneal fat tissue, increasing the aortomesenteric angle and reducing obstructive symptoms [17,18]. With these measures and advances in both enteral and parenteral nutrition, the need for operative intervention has decreased from 70% to 14% from 1974 to 2006 [13].

Operative management is indicated only when conservative management fails [17]. Operative treatments include gastrojejunostomy, Strong’s procedure, and duodenojejunostomy. Gastrojejunostomy provides gastric decompression but fails to completely decompress the duodenum, leading to post-operative complications such as blind loop syndrome and persistence of symptoms [19]. In Strong’s procedure, the ligament of Treitz is divided to mobilize and derotate the duodenum [20]. While Strong’s procedure is not technically difficult and does not involve bowel anastomosis, it has also been shown to have a relatively high failure rate of up to 25% [4]. In fact, our patient had the ligament of Treitz divided and the third portion of his duodenum fully dissected as part of his liposarcoma resection. Duodenojejunostomy, which provides gastric and duodenal decompression, is performed most frequently in 69% of surgical cases, with a reported success rate ranging from 80% to 90% [3,6].

## 3. Conclusions

Superior mesenteric artery syndrome is an uncommon phenomenon with post-operative SMA syndrome following intra-abdominal procedures even less prevalent. The unifying theme for most cases of post-operative SMA syndrome is a sudden major rearrangement of intra-abdominal anatomy. We report the first case of post-operative SMA syndrome occurring following resection of a giant retroperitoneal liposarcoma. Post-operative SMA syndrome should be considered in patients intolerant of oral intake following resection of large retroperitoneal tumors, even in the absence of major concomitant visceral resection.

## Data Availability

No new data were created or analyzed in this study. Data sharing is not applicable to this article.
